# Cases Illustrative of Effusion within the Arachnoid Cavity, as a Cause of Sudden Death, after Scarlatina

**Published:** 1850-12

**Authors:** Austin Flint

**Affiliations:** Buffalo


ART. III.—*Cases illustrative of effusion within the Arachnoid Cavity, as a cause of Sudden Death, after Scarlatina.* By AUSTIN FLINT, M. D.

The two following cases appear to the writer to exemplify the occurrence of effusion within the cavity of the arachnoid, as the cause of sudden death. In both cases this conclusion is based solely on the phenomena ex-exhibited before death, there having been no post mortem examinations. They are reported not as containing *evidence* of arachnoid effusion, but as *illustrations* of the points involved in the diagnosis of that event, assuming as correct the principles deduced from observations communicated for the March number (1850) of this Journal. In connection with these cases the attention of the reader is invited to the article just referred to. The first of the subjoined cases was inadvertently omitted in preparing that article. The second case has fallen under observation at a subsequent date. As an occasional, and, in a certain sense, an accidental element in the progress of different diseases, proving the determining cause of a fatal termination, the occurrence of this lesion has, in the opinion of the writer, in several instances been apparent in cases that have been observed since that article was published ; and, if the views of the writer on this subject be correct, every practitioner will have no difficulty in finding illustrations. The two cases now reported, however, appear to present the occurrence of the lesion isolated in a striking manner from other obvious elements of disease, and to exemplify, in consequence, more vividly the circumstances involved in the diagnosis.

CASE 1. June 15th, 1847, I was requested, at night, to visit a patient in consultation with Dr. J. B. Pride, of this city. I found, on arriving at the house, the patient, a young girl aged 18, unconscious, with tracheal rattle, cold extremities, and evidently in *articulo mortis. The pulse, however, had considerable volume* (the frequency is not recorded.) *The respiration was irregular and convulsive, several respirations preceding each other, followed by a long interval.* She lived about two hours after I first saw her. Dr. Pride gave me the following history:—She has been in delicate health for some time. On the day but one preceding her death, she was attacked with Scarlatina. The disease appeared to be mild. There was moderate Pharyngitis, and the eruption was abundant on the day and evening of her death. Dr. P. had visited her at ten o'clock on that evening, and found her sitting up. She reported pretty comfortable, and anticipated feeling much better the next day. Her pulse was considerably accelerated, enumerating 120. There existed much pruritus of the skin.

Dr. P. prescribed a few grains of Dover's powder. At eleven o'clock, her mother, who had not retired, observing that shortly after conversing she became unconscious, with the eye-balls upturned, became greatly alarmed, and sent in haste for Dr. P., who resided but a few rods distant. On the arrival of Dr. P., he found her awake, declaring she was comfortable, and expressing surprise that her friends were alarmed. She was not sensible of any unpleasant change in the symptoms. Dr. P. remained to observe the patient for a time. After talking jocosely for a few moments, ridiculing the fears of her mother, she dropped asleep, and Dr. P. noticed that her respiration became heavy and stertorous. He was told, however, that this was not unusual. She awoke several times spontaneously, and remarked herself on her audible breathing. At length Dr. P. observed a slight convulsive tremor, and rolling upward of the eye balls. He immediately attempted to arouse her, and with partial success; but she speedily lapsed into a state of complete insensibility. *The respiration now became irregular and rattling as it was when I saw her,* which was about half an hour after the development of the coma. *Deglutition was impossible.* Dr. P. had resorted to the application of sheets dipped in hot water, and sinapisms, but with no benefit.

CASE 2. A child of Mr. D. S. R., between 4 and 5 years of age, had mild scarlatina, in June, 1850. It was accompanied by moderate pharyngitis, and considerable enlargement of the submaxillary glands. The child convalesced in a few days so as to be about the house, the submaxillary glands, however, remaining swelled, and my visits were discontinued. Subsequently I was called to prescribe for the enlarged glands, and I directed the Iod. Potassii. The child continued pretty well, appetite good, was out of doors, and, owing to the feeble, health of the mother, did not, perhaps, receive that degree of care, as regards exposure, etc., which otherwise would have been bestowed.

On the 7th July, the father informed me, at a casual meeting in the street, that the limbs, abdomen, and face of the child appeared bloated. Suspecting that the renal function might be at fault, I requested him to bring me a vial of the urine, which he did on the following morning. On testing the specimen with nitric acid, a copious deposit of albumen was was thrown down. At evening of the same day I was requested to visit the child. He had continued to be up and about, playing out of doors; had been bright during the day, and had accompanied his parents in the afternoon, in taking a ride. The abdomen and limbs were enlarged in bulk, but the latter did not pit on pressure. The face also se[illegible]

swelled. *The respiration, since evening, had become much accelerated, and was now very rapid, panting.* I thought effusion into the chest might have taken place, but, on percussion, no physical evidence appeared of this as the cause of the disordered respiration. *The child did not appear to suffer from dyspnœa, although he was extremely restless.* His muscular strength was not prostrated, being able to sit up. The pulse was much accelerated, and tolerably developed. I directed a warm half bath, and a solution of the sulphate of magnesia in hourly doses, until free cathartic operation. I am free to state that I did not anticipate the sequel. The deglutition was not impaired.

In about an hour afterward, I was summoned in haste, and on my arrival at the house, I found the patient already dead.

The disordered respiration had continued unabated, but not increased, until a few moments before the messenger was despatched, when it suddenly became extreme, and death took place in less than half an hour after this change occurred. To use the father's expression, the child seemed to "*choke to death.*" *A neighbor informed me, of her own accord, without any questions, that she felt the heart beat at the precordial region, after the respiration had ceased.*

*Remarks.* I have italicised, in the foregoing histories, the passages which relate to points of special importance in the diagnosis. These points are disordered respiration, eventuating in apnœa, the action of the heart in the first case, persisting and well developed after the patient was evidently moribund; and, in the second case, continuing after the cessation of the function of respiration. The circumstances upon which the diagnosis is based in these two cases, then, are as follows:

In the first case, coma suddenly and unexpectedly developed, not preceded by any aberration of the intellect, nor by any cerebral symptoms, but for a short period by heavy and stertorous respiration during sleep; death by apnœa, the deglutition affected simultaneously with the fatal disorder of the respiration. This group of circumstances, as it seems to the writer, denotes that the morbid agency which proved the immediate cause of death was, in the first place, cerebral, and, in the second place, produced its fatal consequences by suspending the functions of the medulla oblongata—that portion of the cerebro-spinal centre which sustains direct relations with the movements of respiration and deglutition. The same group of phenomena has been shown to be produced by haemorrhagic effusion into the arachnoid cavity, (see No. of Journal for March, 1850, for cases.) Effusion of serum into the same cavity, it would be fairly inferred by analogy, would produce the same phenomena, and be followed by the same result. But the presence of serous effusion in this cavity has been proved to exist in cases in which sudden death, under similar circumstances, has taken place. (See ibid.)

In the second case, notably disordered respiration, suddenly developed, without coma, and deglutition preserved ; the disorder of respiration suddenly increased, producing apnœa and sudden death, the impulse of the heart persisting after the cessation of respiration. An analysis of this collection of phenomena, as in the first case, leads us to the medulla oblongata as the source of the fatal apnœa.

It is to be observed, in this case, that the embarrassment in respiration, although great, was unattended by dyspnœa, or a painful consciousness of a defective performance of this function. This is a distinctive feature of disordered respiration, tending to apnœa, proceeding from compression of the medulla oblongata. The sense of the want of respiration (besoin de respirer) is impaired, in proportion to the diminished motive acts for carrying on the function—hence, the absence of that extreme distress attending defective hæmatosis from affections of the lungs or heart. This is a diagnostic point to be borne in mind.

In the last case, the existence of albuminaria, accompanied by the accumulation of serum in the abdomen and cellular tissue, favors the supposition of serous effusion into the arachnoid.

In both cases the fatal affection might with propriety be nosologically included under the term apoplexy ; and, if the pathology of the writer be correct, they are probably instances of serous apoplexy.

The cases are interesting from the fact of the sudden death occurring in connection with scarlatina.

They are reported, with the foregoing few remarks, of necessity hastily penned, with a view to solicit the attention of pathological observers to the subject of serous effusion within the arachnoid cavity as a cause of sudden death, and as a mode in which a fatal termination occurs in various diseases.

Buffalo, November 18th, 1850.

